# Poly(butylene succinate-*co*-ε-caprolactone) Copolyesters: Enzymatic Synthesis in Bulk and Thermal Properties

**DOI:** 10.3390/polym13162679

**Published:** 2021-08-11

**Authors:** María Núñez, Sebastián Muñoz-Guerra, Antxon Martínez de Ilarduya

**Affiliations:** Departament d’Enginyeria Química, Universitat Politècnica de Catalunya, ETSEIB, Diagonal 647, 08028 Barcelona, Spain; maria.nunez.gallego@gmail.com (M.N.); sebastian.munoz@upc.edu (S.M.-G.)

**Keywords:** aliphatic polyesters, poly(butylene succinate-*co*-ε-caprolactone), enzymatic synthesis, CALB lipase, molecular weights, microstructure, thermal properties

## Abstract

This work explores for the first time the enzymatic synthesis of poly(butylene-*co*-ε-caprolactone) (PBSCL) copolyesters in bulk using commercially available monomers (dimethyl succinate (DMS), 1,4-butanediol (BD), and ε-caprolactone (CL)). A preliminary kinetic study was carried out which demonstrated the higher reactivity of DMS over CL in the condensation/ring opening polymerization reaction, catalyzed by *Candida antarctica* lipase B. PBSCL copolyesters were obtained with high molecular weights and a random microstructure, as determined by ^13^C NMR. They were thermally stable up to 300 °C, with thermal stability increasing with the content of CL in the copolyester. All of them were semicrystalline, with melting temperatures and enthalpies decreasing up to the eutectic point observed at intermediate compositions, and glass transition temperatures decreasing with the content of CL in the copolyester. The use of CALB provided copolyesters free from toxic metallic catalyst, which is very useful if the polymer is intended to be used for biomedical applications.

## 1. Introduction

Aliphatic polyesters have been recognized as environmentally friendly polymers. They present biodegradable properties and a great biocompatibility, which places them in a privileged position among bio-based polymers [1]. They are used for several green and biodegradable applications related to biomedicine and pharmaceutical technology, such as tissue engineering, drug delivery systems, sutures, orthopedic devices, and implants, among others [2,3,4,5,6,7]. 

Two aliphatic polyesters of special interest are poly(butylene succinate) (PBS) and poly(ε-caprolactone) (PCL). PBS is a biodegradable and bio-based polyester obtained by melt polycondensation of dimethyl succinate (DMS) or succinic acid (SA) and 1,4-butanediol (BD). It is a semicrystalline polymer with mechanical properties comparable to isotactic poly(propylene). It has a melting point of 114 °C and a glass transition temperature of −32 °C [8,9,10]. On the other hand, PCL is obtained by ring opening polymerization of ε-caprolactone (CL). This semicrystalline polyester has a melting point of 65 °C and a glass transition temperature of −60 °C. As for the case of PBS, the biodegradability and biocompatibility of PCL makes it attractive for biomedical applications [11,12,13,14]. PCL shows several critical drawbacks, such as poor thermal properties and a poor chemical solvent resistance. Copolymerization presents a great solution to overcome these limitations [8,15,16,17]. It offers a wider range of possible combinations of properties, the exact balance of which can be tuned through the copolymer composition. These polyesters and copolyesters are usually produced using metallic and organometallic catalysts such as titanium (IV) isopropoxide (TIP), aluminum alkoxides, and tin octoate, which are difficult to remove from the final polymer, making the material too toxic for use in the biomedical field [18,19,20]. 

The latest developments in the field of polycondensation and ring opening polymerization show that lipases provide an excellent opportunity to achieve the “green polymer chemistry” goal (that is, finding chemistry processes that do not harm the environment). Enzymatic catalyzed reactions present several advantageous characteristics, which entail, among others, the use of mild reaction conditions which avoids the generation of undesirable side products, high enantio- or regio-selectivity, the use of non-toxic metallic reagents, and the possibility for the enzyme to be reused several times, contributing to global sustainability [21,22,23]. One drawback is that the enzyme has to be removed from the final polymer in order to avoid its enzymatic degradation. In fact, CALB has been used for the synthesis of both polyesters (PBS and PCL) [24,25,26,27,28,29]. Although poly(butylene succinate-*co*-ε-caprolactone) (PBSCL) copolyesters have already been synthesized using organometallic catalyst [30,31,32], an attempt to produce them using enzymes by ring opening polymerization of CL and butylene succinate cyclic oligoesters provided low molecular weight copolymers, which could restrict their properties and consequently their applications [33].

In an effort to improve those drawbacks, an enzymatic polymerization of PBSCL copolyesters using CALB catalyst from commercial monomers (CL, BD, and DMS) is proposed, which makes the reaction much simpler because the step of the formation of cycles is avoided. The use of this catalyst avoids the presence of metallic and organometallic compounds in the final polymer, which is an added benefit if the polymers are to be used for biomedical applications such as scaffolds, excipients for drug delivery systems [34], or sutures. A preliminary study of the kinetics of the reaction and a full characterization of the structure and thermal properties of the eco-friendly synthesized polymers are addressed.

## 2. Materials and Methods

The reagents dimethyl succinate (DMS) for synthesis was from Merck (Darmstadt, Germany), and 1,4-butanediol (99%) (BD) and ε-caprolactone (97%) (CL), both purchased from Sigma-Aldrich (St. Louis, MO, USA), were used without further purification. 1,4-butanediol was treated with molecular sieves for 24 h. The enzyme *Candida antartica* lipase B (Novozyme-435, CALB) was kindly donated by Novozymes (Bagsværd, Denmark). The reported activity of the enzyme is 10,000 PLU/g. It was stored in a refrigerator and dried in a vacuum desiccator at 50 °C for 24 h before use. Solvents used for removing the enzymes, such as chloroform or tetrahydrofuran, purchased from Sigma-Aldrich, were used without further purification.

FTIR spectra were recorded using a Perkin Elmer-Frontier (Waltham, MA, USA). spectrophotometer with a UATR accessory. The spectral width was from 4000 to 450 cm^−1^, accumulating 8 scans for each run. ^1^H and ^13^C NMR spectra were recorded on a Bruker AMX-300 spectrometer (Billerica, MA, USA) at 25 °C. The spectrometer operated at 300.1 MHz for ^1^H and at 75.5 MHz for ^13^C spectra. The samples, approximately 10–40 mg, were dissolved in 1 mL of a deuterated chloroform (CDCl_3_) solution. The spectra were internally referenced to TMS. A total of 32 or 128 scans were acquired for ^1^H and between 1000 and 10,000 scans were recorded for ^13^C.

The molecular weights of the prepared copolyesters were measured in Waters GPC equipment (Foster City, CA, USA) with RI and UV detectors. A 1–2 mg sample was dissolved in 1 mL of 1,1,1,3,3,3-hexafluoro-2-propanol and was injected and chromatographed with a flow of 0.5 mL/min. HR5E and HR2 Waters linear Styragel columns (7.8 × 300 mm, pore 10^3^–10^4^ Å) packed with crosslinked polystyrene and protected with a precolumn were used. Poly(methyl metacrylate) (PMMA) standards with narrow molecular weight distributions were employed to generate a calibration curve. Intrinsic viscosities of polyesters were measured in an Anton Paar AMWn Automated Micro Viscosimeter (Graz, Austria). Intrinsic viscosities of polyesters dissolved in chloroform were measured at 25 °C. Moreover, the average molecular weight was calculated with the Mark–Houwink–Sakurada relation [35].

$$\left[\eta \right]=6.4\times 10^{-4}\cdot {\bar{M}}_{n}^{0.67}$$

Thermogravimetric (TGA) studies were carried out using Star System (Mettler Toledo (Columbus, OH, USA)) equipment. Samples were heated from 30 °C to 600 °C at 10 °C/min. The experiments were carried out under a nitrogen flow of 20 mL/min in order to maintain an inert atmosphere. DSC thermograms were obtained on a Perkin Elmer DSC Pyris 1. Thermograms were obtained from approximately 5 mg samples at heating and cooling rates of 10 °C/min under a nitrogen flow of 20 mL/min. The standards used for temperature and enthalpy calibrations were indium and zinc. The glass transition temperature (*T*_g_) was taken at the inflection point from hot melt-quenched samples heated at 20 °C/min 30 °C above their melting temperatures and the melting temperature (*T*_m_) was obtained from the endothermic peak observed in the second heating scan.

### 2.1. Synthesis of PBS_x_CL_y_ Copolyesters

Poly(butylene succinate-*co*-ε-caprolactone) (PBSCL) was synthesized through enzymatic ring opening polymerization/polycondensation using *Candida antarctica* lipase B as a catalyst. The nomenclature for the copolymer is PBS_x_CL_y_, where *x* is the % of butylene succinate and *y* is the % of the caprolactone feed molar ratio. The polymerization was carried out in bulk, at different molar ratios (30/70, 50/50, and 70/30) and the parent homopolymers were synthesized as well. A three-necked reactor with a mechanical stirrer, a nitrogen inlet, and a vacuum distillation outlet was charged with different amounts of DMS, BD, and CL. Then, the predetermined amount of immobilized CALB (10% in bulk of the total amount of monomers) was added. This concentration of enzyme was selected by taking into account previous studies of ROP/polycondensation in bulk using this enzyme [36]. An excess of 1% of BD over DMS was used in order to achieve oligomer chains with hydroxyl end groups. During the process, the reactor was submerged in an oil bath to maintain the reaction temperature at 90 °C or 100 °C, depending on composition, and continuously stirred at 30 rpm to obtain a homogeneous mixture. The reactions were carried out in two steps. In the first step, transesterification and ROP reactions were carried out under a very low nitrogen flow to enhance the reaction through the formation of oligomers and to remove the methanol byproduct, taking approximately 4–6 h. After that, in the second step, the polycondensation reaction was performed for 7–24 h at the same temperature under 0.03–0.05 mbar of vacuum in order to increase the molecular weight of the oligomers produced in the first step. Finally, when the reaction ended, the polymer mass was dissolved in chloroform, the enzyme removed by filtration, and the solvent evaporated at room temperature. The same methodology was followed to synthesize PCL and PBS homopolymers, although in the case of PBS, the polycondensation temperature had to be increased to 110–120 °C to avoid polymer crystallization.

In order to remove the remaining solvent and the humidity of the samples, all samples were dried at 50 °C for 24 h under vacuum before carrying out the characterization methods. 

### 2.2. Procedure for the Kinetic (Michaelis–Menten) Study of the Reaction of DMS and CL with Benzyl Alcohol

Reactions of DMS, CL, and an equimolar mixture of these two monomers with benzyl alcohol were carried out in NMR tubes, using deuterated toluene as a solvent and TMS as am internal reference. First, 5, 10, and 20 mg of CL or DMS or CL/DMS (1/1 molar ratio) and 94.9 mg of benzyl alcohol were dissolved in 1 mL of deuterated solvent and added to an NMR tube. After that, 2 mg of CALB were added and samples inserted in the NMR equipment which was thermostatted at 70 °C. ^1^H NMR spectra were collected at 5, 20, 60, 90, and 120 min of reaction. After each NMR acquisition (32 scans), the NMR tubes were taken out and shaken and placed into the NMR equipment again, following a similar procedure to the one reported by Mei et al. [27]. Initial rates were calculated from the inverse of the slope of CL or DMS disappearance vs. time of reaction. 

## 3. Results and Discussion

**Figure 1:**
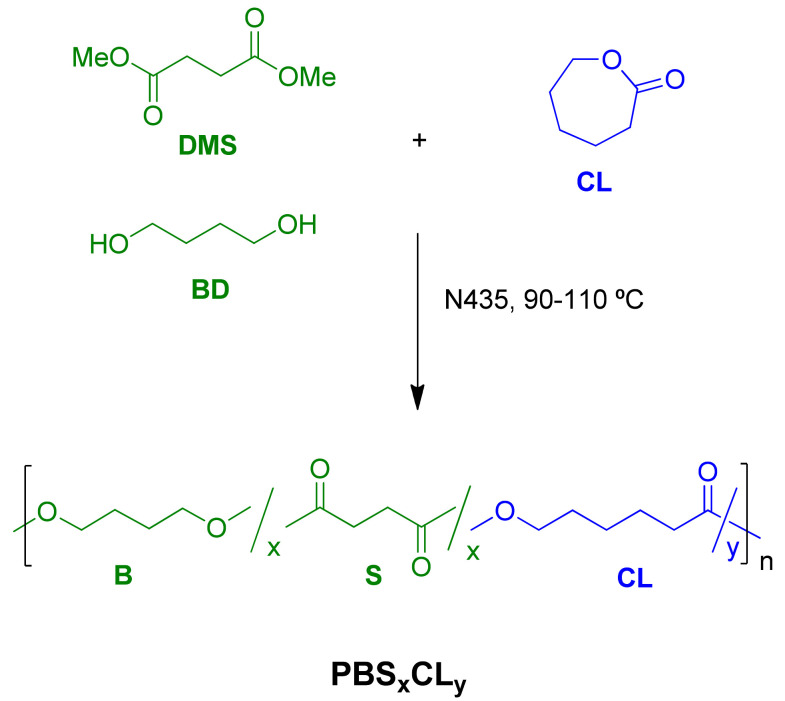
Synthetic route to PBS_x_CL_y_ copolyesters.

The synthesis of poly(butylene succinate-*co*-ε-caprolactone) copolyesters was carried out in two steps. The first step is based on the transesterification and ROP reactions, in which oligomers of PBSCL are formed at about 90 °C. This part of the reaction took approximately 4–6 h. In the second step, the polycondensation took place under vacuum for 7–24 h. In the case of PBS, both steps took much more time than was expected. The transesterification took approximately 12 h while the polycondensation was carried out in 24 h, most likely due to the high melt viscosity of the polymer caused by its higher *T*_g_. Then, for this polyester, the temperature in the second step had to be increased to 110 °C to prevent polymer crystallization. The methodology for the preparation of PBS_x_CL_y_ copolyesters is depicted in Figure 1.

**Figure 2:**
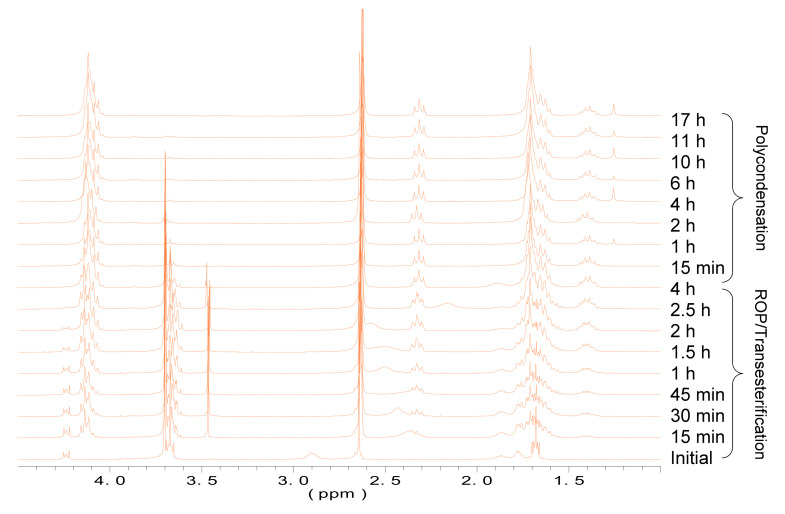
Evolution of ^1^H-NMR spectra of PBS_70_CL_30_ copolyester synthesis with reaction time. The initial sample was recorded without CALB added.

^1^H-NMR was used to follow the reaction. Figure 2 shows the evolution of the enzymatic polymerization of PBS_70_CL_30_ copolyester as a representative example.

By means of some signals of the ^1^H NMR spectrum, it was possible to follow and control the reaction during the synthesis of all copolyesters. The first nine spectra represent the ROP/transesterification step, which in this case took approximately 4 h. Then, the following eight spectra show the reaction evolution during the polycondensation step, which was carried out under vacuum for 17 h.

Considering the spectra from downfield to upfield shifts, the first multiplet at 4.3–4.2 ppm corresponds to the oxymethylene protons of unreacted CL. As expected, this signal decreases during the first step of the reaction, due to the ring opening polymerization. The next peaks (4.2–4.1 ppm) grow in intensity according to the evolution of the reaction because they are due to the esterified oxymethylenes of CL and BD. The release of methanol and the decrease in the signal due to methyl groups of the dimethyl succinate can be observed at 3.5 and 3.7 ppm, respectively. The signals of the rest of methylenes of BS and CL appear between 2.7 and 1.4 ppm, and they are upfield shifted from the values of the unreacted CL and downfield shifted for the unreacted BD. In addition, and with the intention to demonstrate that the enzyme plays a crucial role in the reaction, the monomers without it were brought under the same reaction condition (T = 90 °C) for 24 h. As can be observed in Figure S1 of the Supplementary Information for PBS_70_CL_30_ copolymer, no reaction took place under these conditions.

**Figure 3:**
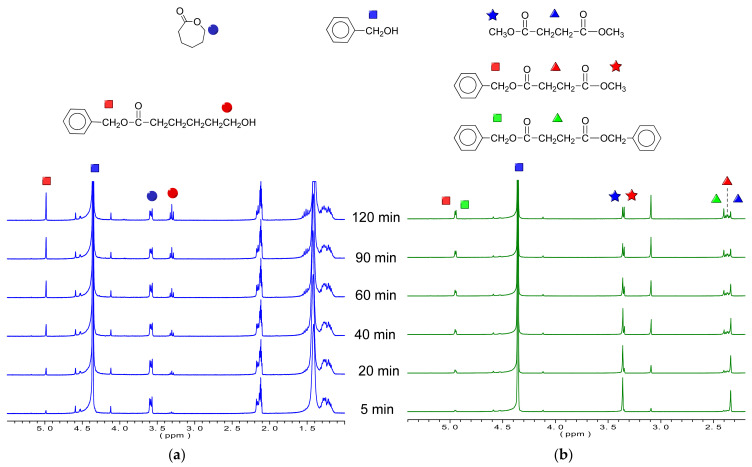
^1^H NMR spectra of the reaction of BnOH with CL (**a**) and DMS (**b**) in the presence of CALB at 70 °C in deuterated toluene. CL and DMS substrate concentration kept at 5 mg/mL.

In order to compare the affinity of CL and DMS to the active site of CALB enzyme, a preliminary kinetic study of these two monomers in solution was carried out in the presence of benzyl alcohol (BnOH). The use of this initiating alcohol (primary as well as BD) allowed for following the reaction more easily by ^1^H-NMR. Different substrate concentrations were used, while the amounts of enzyme and BnOH were kept constant. As an example, the reactions of CL and DMS with BnOH in the presence of CALB are compared in Figure 3. From the initial rates of disappearance of CL and DMS at different substrate concentrations, the Michaelis–Menten constant (*K_m_*) as well as the maximum rate of reaction *V*_max_ were determined using a Lineweaver–Burk plot (Figure S2 of Supplementary Information). These values are depicted in Table 1. As can be observed, DMS displayed a lower *K*_m_, indicating a higher affinity of this substrate for the CALB. Additionally, the *V*_max_ observed is higher for this substrate. It can be concluded, then, that DMS is more reactive under these conditions with values of *k*_cat_/*K*_m_ that are more than twofold the values observed for CL.

Table 2 summarizes the average molar masses of PBSCL copolyesters (both in number and weight) and dispersities, which were estimated by GPC (Figure S3 of Supplementary Information) and by NMR. Furthermore, intrinsic viscosity values are shown and the estimated value of *M*_n_ for PBS using the corresponding Mark–Houwink equation [35] is depicted. In general, there is a good agreement between the *M*_n_ values obtained by these techniques. Weight average molecular weights oscillate between 21,800 and 37,500 and are higher than the values obtained previously by us from CL and cyclic butylene succinate oligomers [33]. The dispersities vary between 2.6 and 3.9, showing no apparent correlation with copolymer composition. These high values of dispersities observed could be caused by the high viscosity of the melt at the temperatures used in the reaction that prevented adequate stirring of the reaction mass. Finally, it can be observed that intrinsic viscosity is tightly correlated with molecular weight, that is, it increases with the CL content. These values oscillate from 0.25 to 0.53 dL/g.

**Figure 4:**
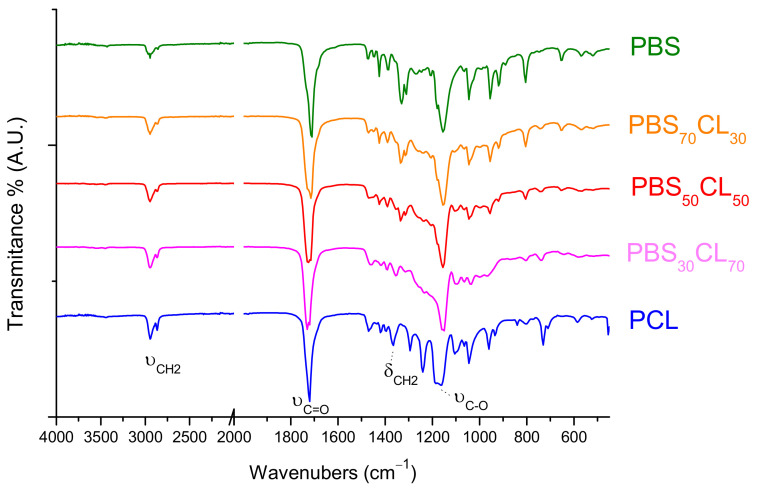
FTIR spectra of PBS, PCL, and PBS_x_CL_y_ copolyesters.

Figure 4 shows the infrared spectra of the copolymers where the representative absorptions of the functional groups of each comonomeric unit can be distinguished. The characteristic ester-stretching (C=O) absorption appears at 1721 cm^−1^ for PCL, and it is shifted to lower frequencies for PBS, which appears at 1712 cm^−1^. Absorption bands due to C-O stretching are observed at 1162 and 1152 cm^−1^ for PCL and PBS, respectively. For PBS_x_CL_y_ copolyesters, both carbonyl stretching bands are observed, with the intensity of the 1721 cm^−1^ absorption increasing as the content of CL increases in the copolymer. Additionally, for PBS_x_CL_y_ copolyesters, a broad band centered at 1729 cm^−1^ is observed, which is attributed to both carbonyl stretching absorptions in the amorphous phase.

The C-H stretching bands appear in the wide range between 3000 and 2840 cm^−1^, giving a weak signal due to the low dipolar moment of these functional groups. As was expected, the relative intensity of these absorptions increases with the content of CL in the copolymer.

**Figure 5:**
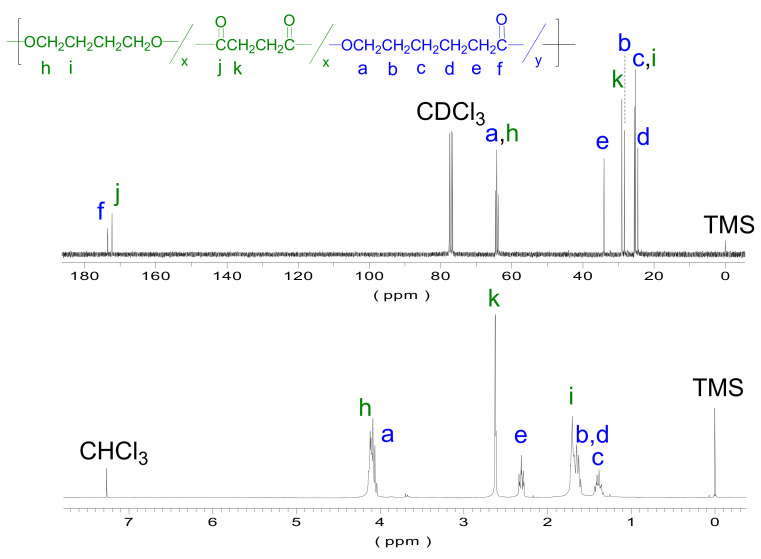
^1^H (bottom) and ^13^C (top) NMR spectra of PBS_50_CL_50_ recorded in CDCl_3_ with peak assignments.

NMR was further used for determining the copolyester composition and microstructure of the copolymers obtained. Figure 5 depicts the ^1^H and ^13^C NMR spectra of PBS_50_CL_50_. Spectra of all series are included in Figures S4 and S5 of the Supplementary Information.

Three groups of peaks can be differentiated: methylenes next to an oxygen atom (peaks *h* and *a* in Figure 5, bottom), next to a carbonyl (peaks *k* and *e* in Figure 5, bottom), and next to another methylene group (peaks from *i* to *c* in Figure 5, bottom). The signal at 4.0–3.9 ppm corresponds to a methylene next to an oxygen and varies from a multiplet in PBS to a triplet in PCL, giving different shapes depending on the copolymer composition. The 1.7–1.6 ppm signals show an analogous behavior and occur with the methylene groups and their neighbors, which depend on the composition. Note that the 2.6 ppm (*k*), 2.3 ppm (*e*), and 1.4 ppm (*c*) peaks are signals of the pure succinate or caprolactone units, which allow for determining the copolymers’ composition. As can be observed in Table 3, the composition of all copolymers is very close to the initial monomer feeding. The results show that the variation between the feed and the real composition of the polymer varies from 0.8% to 8.5%. This fact can be explained by the occurrence of the loss of volatile compounds during the first and second steps of the reaction.

Analogously, the spectra of the whole ^13^C NMR series are shown in Figure S5 of the Supplementary Information. Three groups of signals can be distinguished. The first group appears at approximately 170 ppm, showing the C=O signals corresponding to ester bonds from CL units and the succinate part from BS units. The signal at 174 ppm corresponds to the ester bond from CL units, whilst at 172 ppm the signal of succinate units appears. As can be seen, the intensity of these peaks varies depending on the copolymer composition. The second group corresponds to the methylenes located next to oxygen, i.e., present in both CL and BS units. These O-CH_2_ signals appear at around 64 ppm and, in some cases, they are split due to sequence effects. Lastly, the third group of peaks appears between 35 and 25 ppm, and corresponds to the methylene carbons attached to different kinds of groups. The first two signals are due to the methylene groups next to carbonyl, and the other ones to inner methylenes.

**Figure 6:**
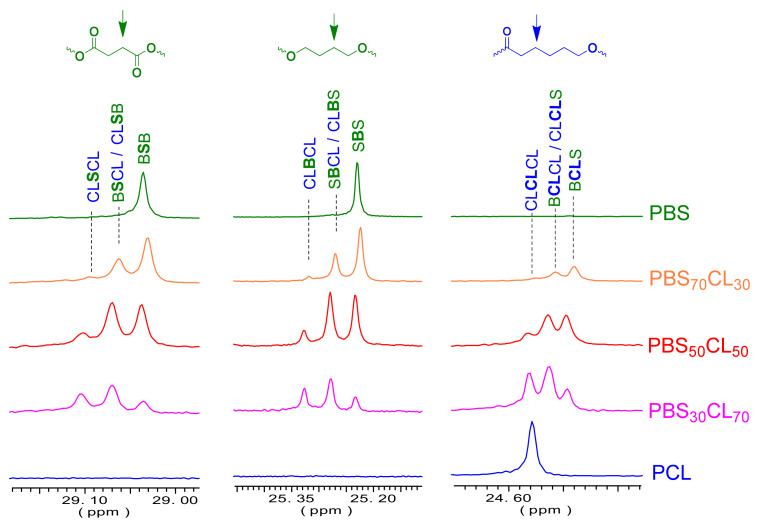
^13^C NMR spectra of PBS, PCL, and PBS_x_CL_y_ copolyesters in the regions where different carbons split due to sequence distribution effects.

Some peaks appeared to be split into three or four peaks (Figure 6) due to sequence distribution effects. As an example, signals from butylene carbons split into tree peaks due to the four butylene-centered triads, CL**B**CL, S**B**CL/CL**B**S, and S**B**S, which were observed and were used for quantification. The relative contents in the four triads for every copolymer composition and the application of the statistical method developed by Tessier et al. [37] led to the determination of the degree of randomness *R* for the PBS_x_CL_y_ copolyesters. 

The microstructure was determined with the ^13^C NMR technique, integrating by deconvolution the split methylene signals from the butylene units located at 25.3 ppm.

The content of B-centered triads allows us to determine the degree of randomness for each copolymer, shown in Table 3. The values of *R* are near one, indicating that these copolymers have a random distribution on comonomeric units. Although initial rates of polymerization indicate that dimethyl succinate is more reactive than ε-caprolactone, transesterification reactions that take place during the polycondensation make the initially blocky copolymers randomize during the reaction process. 

**Figure 7:**
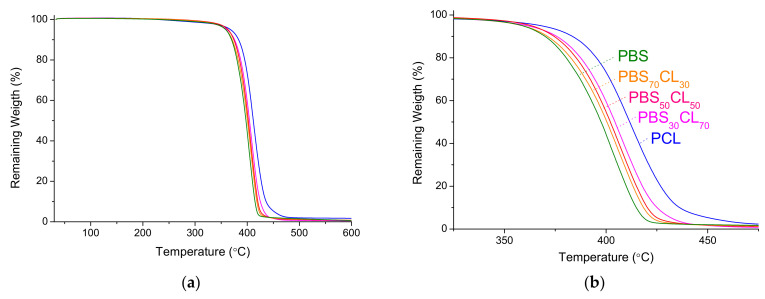
Thermogravimetric traces of PBS, PCL, and PBS_x_CL_y_ copolyesters. (**a**) Full TGA traces; (**b**) expanded region where the thermal degradation with weight loss is observed.

Thermal stability is also well known as an important factor for processing these materials, which will limit their practical application. To evaluate the thermal stability of the copolymers, TGA analyses were carried out from 25 °C to 600 °C. Experiments were performed under a nitrogen flow to keep the atmosphere inert. TGA traces are compared in Figure 7. For a clearer illustration of the observed trend, the 325–475 °C region is expanded in Figure 7b.

These traces depict one decomposition step because both repeating units are structurally similar. Furthermore, the figure shows that thermal stability increases when the CL content increases in the copolymer. The PCL trace shows an analogous behavior until approximately 400 °C, the temperature at which PCL significantly moves away from the other copolymers, a fact that highlights its higher thermal stability. Table 4 summarizes the thermogravimetric data extracted from TGA curves.

**Figure 8:**
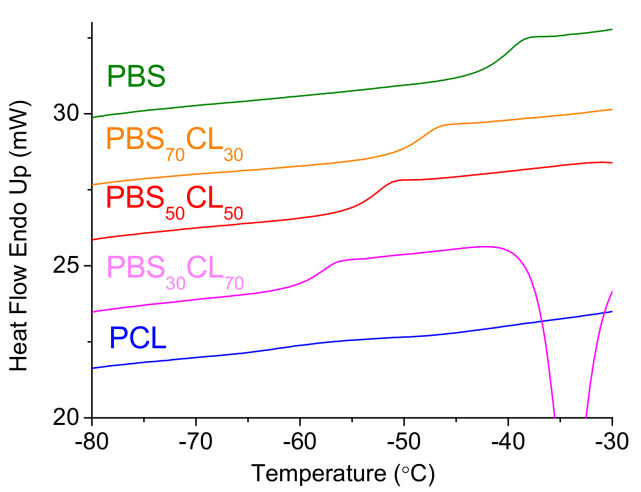
DSC traces of melt-quenched of PBS, PCL, and PBS_x_CL_y_ copolyesters showing the glass transition temperatures.

Thermal transitions for these copolyesters were determined by DSC. Figure 8 depicts the DSC heating thermograms from melt-quenched samples, where the glass transition temperature can be observed as a step in the baseline curve, indicating the change in the heat capacity of the polymer. The glass transition temperatures (*T*_g_) of copolyesters are between the values of two parent homopolymers and vary from −39.9 to −62.1 °C, a clear indication of the random character of these copolymers, as has been observed from ^13^C NMR. PBS has a higher *T*_g_ due to more dipole–dipole interactions that restrict the chain mobility as compared to PCL. In the case of the copolyesters, an incorporation of the CL structural moiety into the PBS polyester chain decreases the crystallinity and considerably decreases the glass transition temperature. Copolymers show a typical Gordon–Taylor evolution with composition, where they have intermediate glass transition temperatures between the pure homopolymers (Figure S6 of Supplementary Information). A value of Gordon–Taylor parameter *k* = 0.46 is intermediate between the values of low molecular weights (*k* = 0.23) and high molecular weights (*k* = 0.63) PBSCL copolyesters are obtained [29].

**Figure 9:**
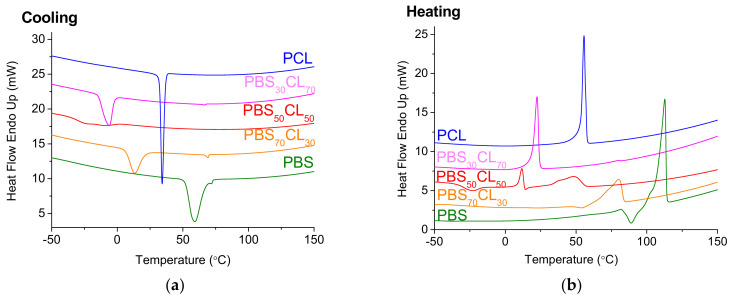
DSC scans of PBS, PCL, and PBS_x_CL_y_ copolyesters. (**a**) Cooling from the melt showing the crystallization exotherms and (**b**) second heating scan showing the melting endotherms.

Figure 9 shows the DSC traces of the copolyesters upon cooling (a) and heating (b). Cooling DSC scans (Figure 9a) depict a crystallization peak during the cooling process in all the polymers, where a very broad exothermic peak is observed for the PBS_50_CL_50_ copolymer. This indicates that the crystallization occurs in the complete range of compositions. For homopolymers and copolymers enriched in one comonomer, it seems that the crystalline structure of the enriched monomer prevails. This behavior has been previously reported by powder X-ray diffraction studies (WAXS and SAXS), where the crystalline structure of PBS was observed for copolyesters enriched in butylene succinate units and the one of PCL for copolyesters is enriched in caprolactone units [32]. In the copolymers with equal content of both comonomers, the two crystalline structures seem to be present, as two exothermic peaks are observed. Both CL and BS repeating units present similar structures, which allow both units to crystallize in different crystal lattices and their content could be modulated by the rate of cooling, as it has been reported by Safari et al. [32], indicating that this eutectic composition presents an isodimorphic crystallization.

The crystallization temperature (*T*_c_) oscillates between 59.2 and −22.9 °C depending on the content of CL in the copolymer. The more CL units in the copolymer, the lower the *T*_c_ up to when an equimolar amount of both comonomers in the copolymer is reached, and then it increases and reaches the value of PCL itself with a crystallization temperature higher than all the copolymers (34.2 °C). These values are in agreement with the ones of PBSCL copolyesters obtained by melt polycondensation using organometallic catalyst [32].

Regarding the melting point, DSC heating scans (Figure 9b) are represented. As happens with crystallization, melting temperatures generally decrease with the content of CL, up to the eutectic point, where two crystallization peaks are observed. Then, it increases up to the value of the melting temperature of PCL. The *T*_m_ decrease reflects a decrease in the crystallite size due to lack of regularity in the copolymer chain. The same trend is observed for the melting enthalpy.

The fact that the melting point and the melting enthalpy of the PBS_50_CL_50_ split into two peaks can be explained by its “symmetrical” structure, as has been described before [32]. Both units present similar structures, with ester groups and the same number of methylenes. This could be the reason why their structures are compatible enough to crystallize together and present two different melting peaks, one from the CL crystallites and the other from the BS crystallites.

The cold crystallization temperature (*T*_cc_) was not detected in the PCL polymer. This is because PCL is a fast crystallization polymer, and it has enough time to crystallize during the cooling process under the non-isothermal crystallization conditions used. The *T*_cc_ thermal parameter varies between −9.5 and −34.0 °C depending on the CL content in each copolymer and, in most cases, shows a direct relationship.

Table 4 summarizes the obtained thermal properties. As can be observed, these parameters strongly depend on the copolymer composition. With respect to the pure homopolymers, the obtained values of *T*_m_ and *T*_g_ are close to the expected values found in the literature [29,32,33], with a deviation of at most 7%.

## 4. Conclusions

This work validates enzymatic polymerization in bulk as a convenient method for the synthesis of PBSCL copolymers. Interestingly, both PBS and PCL had been previously enzymatically polymerized, from commercial starting materials, but not PBSCL copolymers. We succeeded in synthesizing PBSCL copolyesters, as well as PBS and PCL homopolymers, for the entire range of compositions proposed (70/30, 50/50, and 30/70), using the immobilized enzyme *Candida antarctica* lipase B. As is detailed in the Materials and Methods section, the reaction times were significantly longer than those applied in the traditional chemical methods, which usually take a total of 3 h. By comparison, in our case, the transesterification and ROP processes took 4 to 6 h and the polycondensation took 7 to 24 h. Such differences come from the temperatures used for each case. Traditional methods carry out the reaction at higher temperatures, which make the reaction faster. Such temperatures are not applicable for enzymatic synthesis, as they would lead to enzyme denaturalization. The preliminary kinetic study demonstrated that the enzyme has higher affinity towards DMS over CL. The general trend shown by GPC and NMR results is that the polyesters’ molecular weights increased with the content of CL. The structure of the copolymers is equivalent to their chemically derived counterparts. In addition, the NMR sequence distributions demonstrate the random microstructure of these PBSCL copolyesters. One of the goals of synthesizing different compositions (70/30, 50/50, and 30/70) was to observe the possible trade-offs between the thermal properties of PBS and PCL. The TGA and DSC analyses demonstrate that the thermal stability increases and the glass transition temperature decreases with the content of CL in the copolymers. Both the crystallization and melting temperatures and enthalpies decrease for the copolymers up to the eutectic point, which is observed for the (50/50) composition. 

From this perspective, it is considered that these PBSCL copolyesters are a viable and eco-friendly alternative which present good thermal properties and may be prepared with high enough molecular weights under mild reaction conditions, avoiding the use of metallic components. In light of this, these copolymers could be used for biomedical applications, where the presence of toxic compounds even in tiny amounts is a serious health problem. However, because this technique is still in its early stages of development, further research needs to be carried out to bring their cost down before they are widely adopted by industry.

## Figures and Tables

**Table 1:** Michaelis–Menten constants ^1^ of CALB enzyme reaction of CL and DMS with BnOH at 70 °C in toluene solvent.

Substrate	*K*_m_ (mol/L)	*V*_max_ (mol/L·h)
CL	0.158	0.054
DMS	0.091	0.070

^1^ Values determined from the Lineweaver–Burk plots of the reaction of benzyl alcohol with caprolactone (CL) or dimethyl succinate (DMS) in the presence of CALB enzyme.

**Table 2:** Molecular weights and viscosities of PBS, PCL, and PBS_x_CL_y_ copolyesters.

Polyesters	NMR	GPC			Viscosity	
	*M*_n_^1^ (g/mol)	*M*_n_^2^ (g/mol)	*M*_w_^2^ (g/mol)	*Đ* ^2^	[*η*] (dL/g)	*M*_n_^3^ (g/mol)
PBS	8400	8300	21,800	2.6	0.25	7300
PBS_70_CL_30_	8400	9300	36,000	3.9	0.33	-
PBS_50_CL_50_	9100	10,100	27,000	2.7	0.33	-
PBS_30_CL_70_	8600	7100	26,000	3.7	0.39	-
PCL	13,000	9700	37,500	3.9	0.53	-

^1^ Number average molecular weights (*M*_n_) determined from ^1^H-NMR by end group analysis. ^2^ Number and weight average molecular weights and dispersities determined by GPC. ^3^ *M*_n_ determined using the Mark–Houwink equation [35].

**Table 3:** Molar composition and microstructure of PBS and PCL polyesters and PBS_x_CL_y_ copolyesters.

**Polyesters**	**Composition ^1^**		**Microstructure ^2^**			
	**Feed**	**Polyester**	CL**B**CL	CL**B**S	S**B**S	***R***
PBS	100/0	100/0	-	-	-	-
PBS_70_CL_30_	70/30	66.0/34.0	5.4	34.4	60.2	1.07
PBS_50_CL_50_	50/50	50.4/49.6	14.5	46.4	39.1	1,12
PBS_30_CL_70_	30/70	28.9/71.2	32.2	49.7	18.2	1.04
PCL	0/100	0/100	-	-	-	-

^1^ Feed and final molar polyester composition determined by ^1^H NMR. Repeating unit MWs (CL: 146.14 g/mol, BS: 172.2 g/mol); ^2^ microstructure of copolymers determined by ^13^C-NMR. Signals from inner methylenes of butylene units that appear at 25.3 ppm were used for quantification of different triads centered in these units. *R*: degree of randomness calculated using equations taken from reference [37].

**Table 4:** Thermal properties of PBS and PCL polyesters and PBS_x_CL_y_ copolyesters.

Polyesters	TGA ^1^			DSC ^2^				
	^o^*T*_d_^10%^ (°C)	^max^*T*_d_ (°C)	*R*_w_ (%)	*T*_g_ (°C)	*T*_c_ (°C)	*T*_m_ (°C)	*T*_cc_ (°C)	Δ*H*_m_ (J/g)
PBS	370.0	402.9	2.1	−39.9	59.2	112.7	−9.5	74.3
PBS_70_CL_30_	371.2	405.6	1.6	−49.9	13.3	80.0	−14.5	46.7
PBS_50_CL_50_	374.4	406.8	1.8	−53.4	−22.9	11.7/48.2	−19.8	11.0/22.6
PBS_30_CL_70_	375.7	407.4	1.8	−58.4	−6.3	22.4	−34.0	45.8
PCL	378.4	409.3	1.6	−62.1	34.2	55.6	-	53.3

^1^ Onset for 10% (^o^*T*_d_^10%^) and maximum rate (^max^*T*_d_) thermal decomposition temperatures measured by TGA under inert atmosphere. *R*_w_: remaining weight at 600 °C. ^2^ Melting (*T*_m_), crystallization (*T*_c_), cold crystallization (*T*_cc_), and glass transition (*T*_g_) temperatures and melting enthalpy (Δ*H*_m_) measured by DSC.

## Data Availability

Data are contained within the article.

## Supplementary Materials

The following are available online at https://www.mdpi.com/article/10.3390/polym13162679/s1, Figure S1. ^1^H NMR spectra of the reaction of BD with CL and DMS at 90 °C for 24 h in the presence (top) or absence (bottom) of CALB enzyme. No vacuum was applied in the two cases. Figure S2. Lineweaver–Burk plot (1/V versus 1/[S]) for the determination of *K*_m_ and *V*_max_ for the reaction of benzyl alcohol with caprolactone (CL) or dimethyl succinate (DMS) in the presence of CALB enzyme catalyst. Figure S3. GPC chromatograms of PBS, PCL, and PBS_x_CL_y_ copolyesters recorded at 35 °C using HFIP as eluent. Figure S4. ^1^H NMR spectra of PBS, PBS_x_CL_y_, and PCL recorded in CDCl_3_ with peak assignments. Figure S5. ^13^C NMR spectra of PBS, PBS_x_CL_y_, and PCL recorded in CDCl_3_ with peak assignments. Figure S6. Evolution with composition of the glass transition (*T*_g_) of PBS_x_CL_y_ copolyesters.
