# Supplementary material for: Poly(butylene succinate-co-ε-caprolactone) Copolyesters: Enzymatic Synthesis in Bulk and Thermal Properties
Source: Polymers (Basel). 2021 Aug 11;13(16):2679. doi: 10.3390/polym13162679 (PMC8401440; doi:10.3390/polym13162679)
Supplement: Supplementary file 1 [file polymers-13-02679-s001.zip › polymers-1338483-supplementary.pdf]

polymers

MDPI

Supplementary Information

Poly(butylene succinate-*co*-ε-caprolactone) Copolyesters: Enzymatic Synthesis in Bulk and Thermal Properties

María Núñez, Sebastián Muñoz-Guerra and Antxon Martínez de Ilarduya *

Departament d'Enginyeria Química, Universitat Politècnica de Catalunya, ETSEIB, Diagonal 647, 08028 Barcelona, Spain; maria.nunez.gallego@gmail.com (M.N.); sebastian.munoz@upc.edu (S.M.-G.)
* Correspondence: antxon.martinez.de.ilarduia@upc.edu

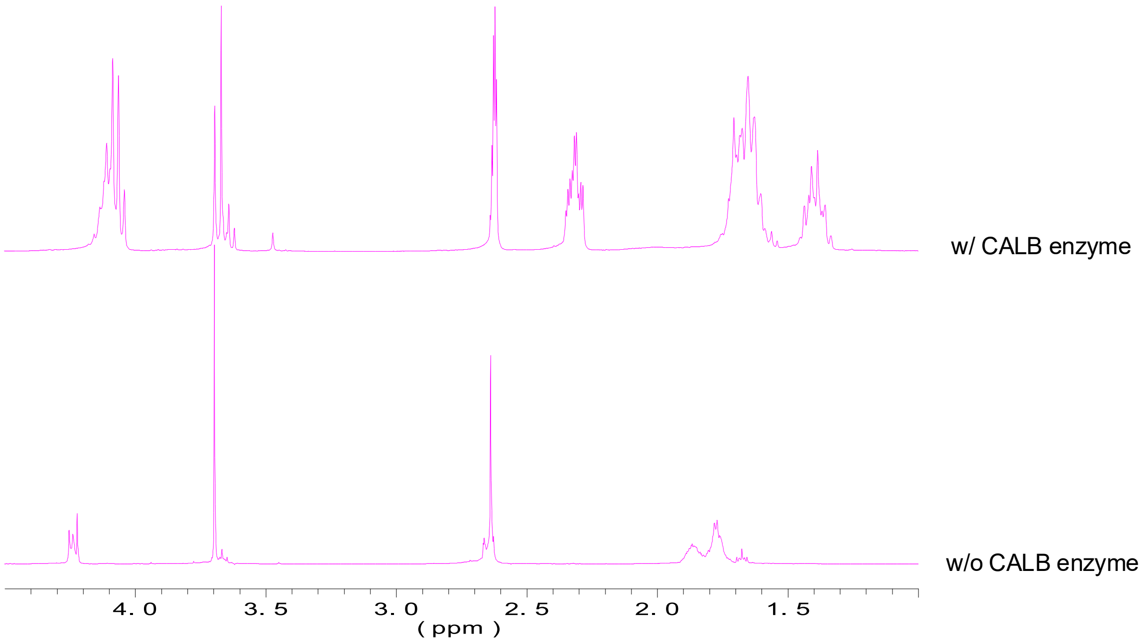

Figure S1. ^{1}H NMR spectra of the reaction of BD with CL and DMS at 90 ºC for 24 hours in the presence (top) or absence (bottom) of CALB enzyme. No vacuum was applied in the two cases.

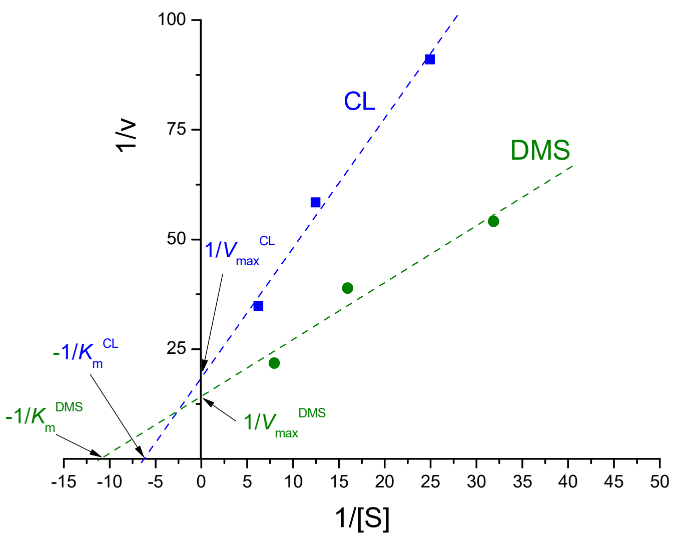

Figure S2. Lineweaver-Burk plot (1/V versus 1/[S]) for the determination of K_m and V_{max} for the reaction of benzyl alcohol with caprolactone (CL) or dimethyl succinate (DMS) in the presence of CALB enzyme.

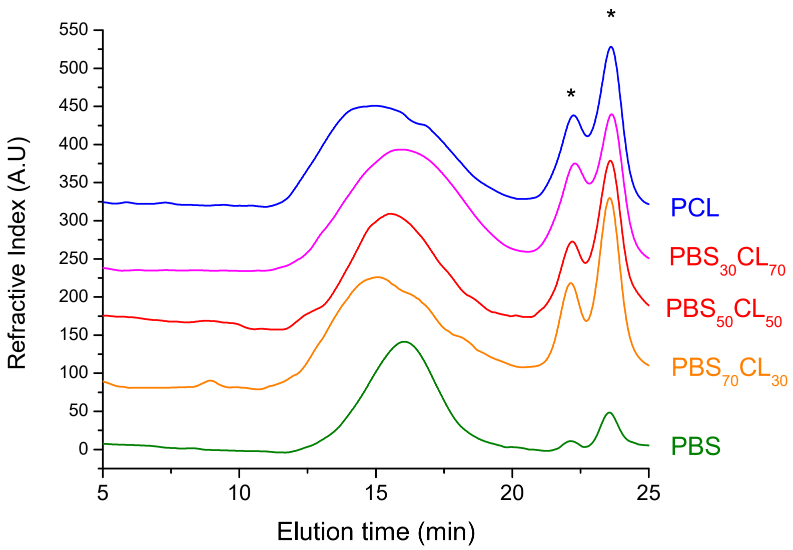

Figure S3. GPC chromatograms of PBS, PCL and PBS_xCL_y copolyesters recorded at 35 ºC using HFIP as eluent. (*) peaks of sodium trifluoroacetate salt and impurity solvents.

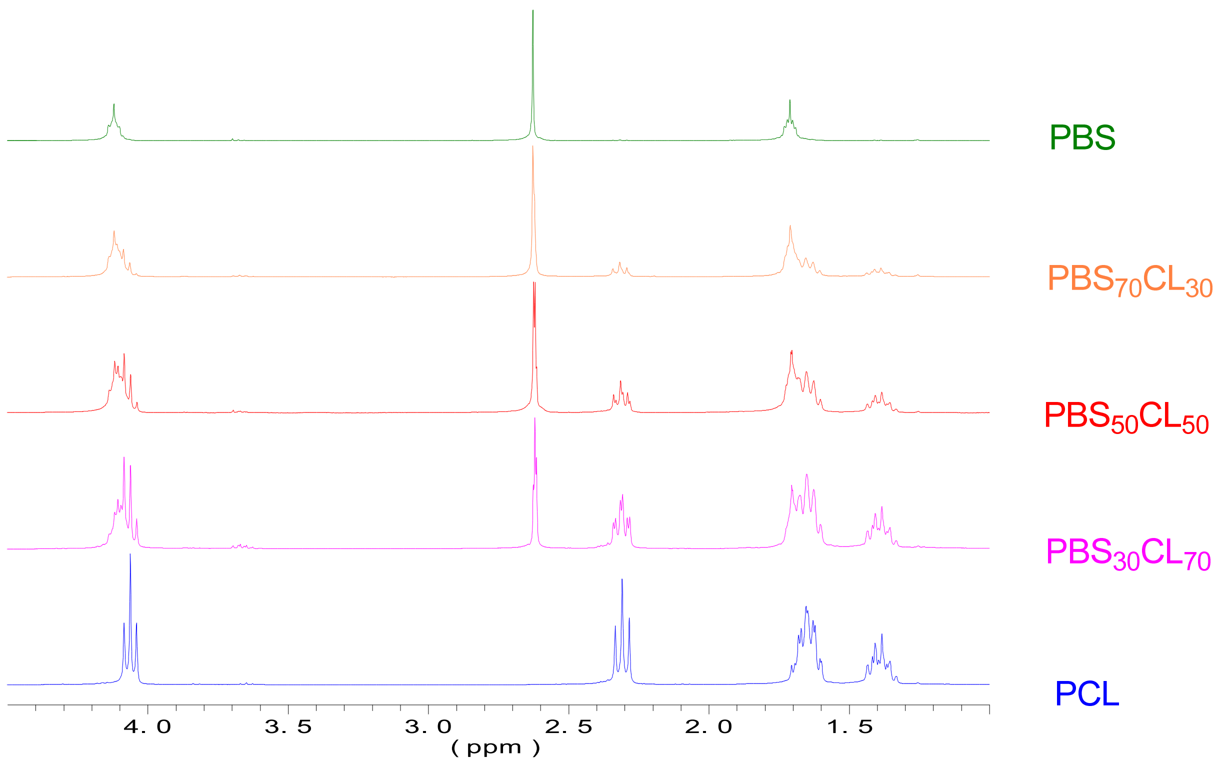

Figure S4. ^{1}H NMR spectra of PBS, PBS_xCL_y and PCL recorded in $CDCl_3$ with peak assignments.

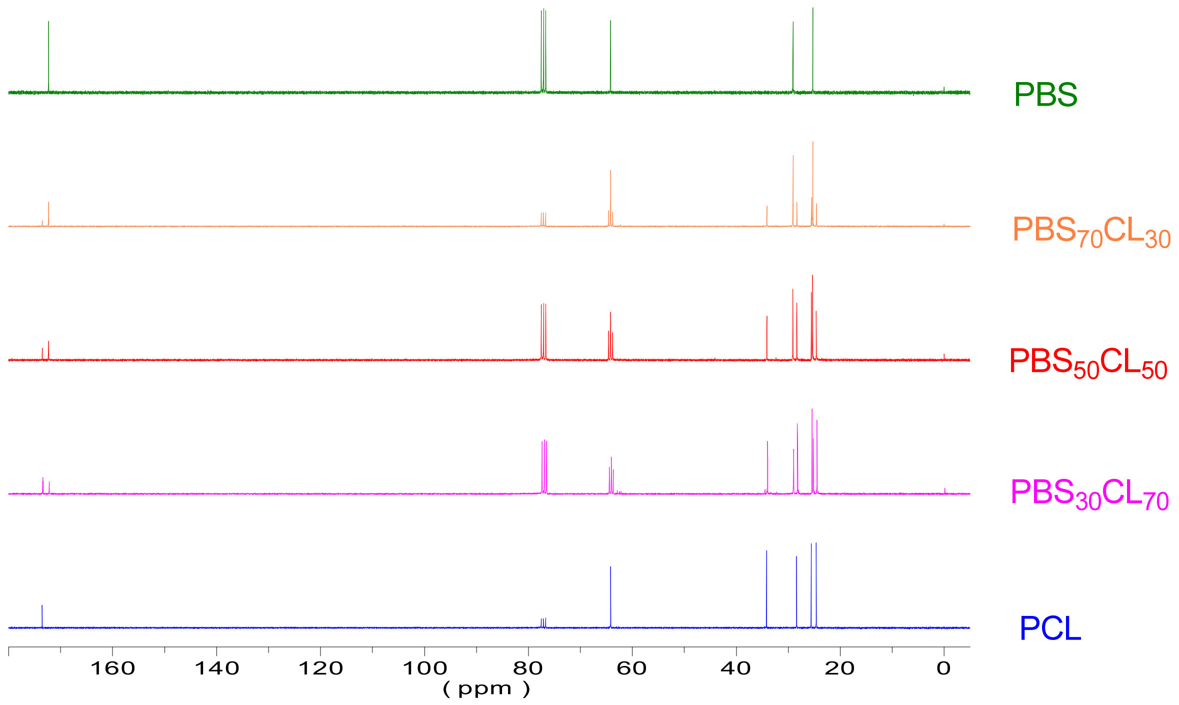

Figure S5. ^{13}C NMR spectra of PBS, and PBS_xCL_y and PCL recorded in $CDCl_3$ with peak assignments.

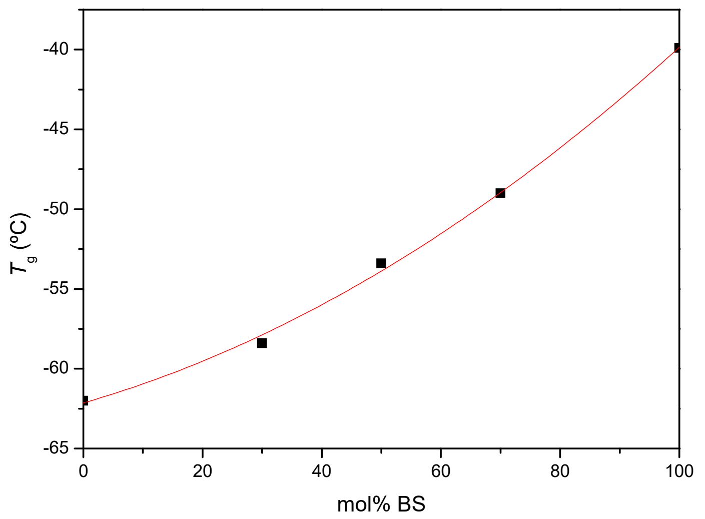

Figure S6. Evolution with composition of the glass transition (T_g) of PBS_xCL_y copolyesters
